# Point-of-Purchase Patterns of Cadmium, Lead, and Other Toxic Elements in *Houttuynia cordata* Thunb. Rhizomes from Retail Markets Across Guizhou, China

**DOI:** 10.3390/foods15193461

**Published:** 2026-09-28

**Authors:** Qingfeng Wang, Xuefeng He, Jia Xu, Dan Wang, Enguo Sheng, Bin Liu, Zhonggen Li

**Affiliations:** 1 School of Ecology, Zunyi Normal College, Pinan Road, Xinpu Town, Zunyi 563002, Chinalizhonggencn@126.com (Z.L.); 2 Zunyi Ecological Environment Monitoring Center, No. 9, Sanxian North Road, Xinpu Town, Zunyi 563002, China

**Keywords:** *Houttuynia cordata* Thunb., cadmium, lead, point-of-purchase variation, health risk assessment

## Abstract

*Houttuynia cordata* Thunb. (HCT) is an edible and medicinal rhizome widely consumed in southwestern China, but its contribution to dietary exposure to toxic trace elements remains insufficiently characterized. Total Hg, As, Cd, Pb, and Cr were quantified in 101 market-level composite samples collected across 76 county-level units of Guizhou Province, and regulatory compliance, point-of-purchase variation, multi-element co-occurrence, and adult dietary exposure were evaluated. Mean fresh-weight concentrations were 4.8, 4.1, 111.5, 291.3, and 6.3 ng/g for Hg, As, Cd, Pb, and Cr, respectively. Pb and Cd exceeded the applicable Chinese maximum limits in 87.1% and 49.5% of samples, respectively, whereas Hg exceeded its limit in 4.0%; no exceedances were observed for As or Cr. FDR-adjusted correlations and principal component analysis indicated limited multi-element co-occurrence rather than a single uniform contamination pattern. The five-element comparative screening HI increased with HCT consumption, with 1, 3, and 17 samples exceeding 1 under the low-, medium-, and high-consumption scenarios, respectively. Cd and Pb were the principal contributors, accounting for mean sample-level contributions of 55.5% and 29.0%, respectively, and together representing 84.5% of the HI. Although the median As contribution was only 4.0%, its 95th percentile reached 49.5%, indicating substantial upper-tail variability. Because Pb was incorporated into the comparative screening HI using a historical normalization benchmark, it was additionally evaluated using an effect-based margin-of-exposure approach. The renal Pb MOE was below 1 in 1.0%, 16.8%, and 32.7% of samples under the three consumption scenarios, respectively. These findings identify Cd and Pb as priority elements in market-sold HCT and support targeted retail surveillance, supply-chain tracing, and refined assessment of samples with elevated As or Pb.

## 1. Introduction

Toxic metal(loid) contaminants in food systems remain a persistent challenge for food safety and public health [1,2]. Elements such as Hg, As, Pb, Cd, and Cr can be released or mobilized through natural geochemical processes, mining and smelting activities, industrial emissions, agricultural inputs, wastewater irrigation, and atmospheric deposition [3,4]. Once introduced into environmental media, these contaminants are difficult to eliminate because they are non-biodegradable and may persist for long periods in soils, sediments, water, and atmospheric particles [5,6]. Their persistence, together with their capacity for biological uptake, creates an important pathway by which environmental contamination can be transferred into the human food chain [7]. Vegetables are particularly relevant matrices for evaluating such transfer [8,9,10,11]. They are consumed frequently, often in relatively large quantities, and many edible tissues develop in close contact with soil or irrigation water. Root and rhizome vegetables may be especially susceptible to soil-derived contamination because their edible parts are directly embedded in the soil environment where metal(loid)s can accumulate [12,13]. Although inhalation and dermal contact may contribute to human exposure in contaminated settings, dietary intake is generally a more continuous and population-wide pathway [14]. Therefore, assessing metal(loid) contamination in commonly consumed vegetables is essential not only for estimating exposure, but also for identifying specific food items and regions where routine consumption may increase health risk.

Among foodborne metal(loid) contaminants, Hg, As, Pb, Cd, and Cr are frequently included in food safety and dietary-risk assessments [15]. Hg is associated with neurotoxicity, particularly in sensitive populations [16]. As exposure through food and drinking water has been linked to increased carcinogenic risk after long-term intake [17]. Pb is strongly associated with neurodevelopmental toxicity, especially in children [18]. Cd can induce adverse effects even at relatively low exposure levels, with the kidney recognized as a critical target organ [19]. Cr is also important in risk assessment because certain chemical forms, particularly Cr(VI), are carcinogenic [20,21]. These different toxicological profiles indicate that a single-element approach may be insufficient for vegetables exposed to multiple contamination sources. A more informative assessment should consider multi-element occurrence, regulatory exceedance, spatial distribution, and cumulative non-carcinogenic risk within the same framework.

*Houttuynia cordata* Thunb. (HCT) is a rhizomatous perennial herb widely distributed in East and Southeast Asia and is particularly common in southern China [22]. It has long been recognized as an edible and medicinal plant, being used not only in traditional medicine but also as a distinctive regional vegetable in daily diets [23]. Pharmacologically, HCT is valued for its bioactive constituents, including volatile oils, flavonoids, and phenolic compounds, which have been associated with anti-inflammatory, antibacterial, antiviral, and immunomodulatory activities [24,25]. In Southwest China, especially in Guizhou, Sichuan, Yunnan, and Chongqing, HCT is deeply embedded in local dietary culture and is frequently consumed as a fresh vegetable, cold dish, seasoning ingredient, or processed food product [26,27]. Although the leaves, stems, and rhizomes are all edible, the rhizome is the most widely consumed part in Guizhou and represents the dominant form sold in local markets. This strong regional consumption preference gives HCT particular significance in food safety assessment. Unlike leafy vegetables, the edible rhizome develops underground and remains in direct contact with soil and irrigation water throughout growth. This growth habit provides a direct pathway for the uptake of bioavailable soil-derived metal(loid)s [28,29] and makes HCT a relevant matrix for food safety assessment.

Guizhou province provides a particularly important setting for such an assessment. This area is characterized by complex karst geology, abundant mineral resources, and a long history of mining and smelting [30,31,32]. These natural and anthropogenic factors can jointly shape the environmental availability of metal(loid)s in agricultural systems. Previous studies have shown that several metals in Guizhou soils exceed local background levels, and that Hg and Cd may contribute disproportionately to ecological risk [31]. The regional significance of Hg is especially notable because Guizhou contains historically important mercury-mining areas, including Tongren and Wuchuan, where mining and ore-roasting activities generated Hg-bearing wastes and long-lasting environmental releases [33,34]. At the same time, Cd and Pb contamination may also be influenced by regional geochemical enrichment, mining-related activities, diffuse industrial inputs, and agricultural practices [35,36]. Therefore, HCT sold in Guizhou markets may integrate contamination signals from both geogenic and anthropogenic sources.

Existing studies have provided evidence that HCT can accumulate multiple toxic elements under field and experimental conditions [23]. In the Wanshan mercury-mining area, HCT showed the highest Hg concentrations in edible rhizomes among 43 crops grown under shared regional exposure, reaching 2.4–1075 ng/g on a fresh-weight basis [37]. Our previous work [27] in the Kaiyang mercury-mining area of Guiyang also indicated elevated Hg in HCT rhizomes, with an average concentration of 42.3 ng/g fresh weight, suggesting that this vegetable may become an important source of Hg exposure for local residents. Beyond Hg, strong Pb accumulation has been reported in cultivated HCT from Ganzi, Sichuan, with high concentrations observed in roots, stems, and leaves [38], and HCT collected from the Beiya Gold Mine area of Yunnan also showed high Pb levels [39]. Pot experiments further demonstrated that HCT can accumulate Cd to levels incompatible with food or medicinal use, with particularly high Cd concentrations in roots under increasing soil Cd exposure [40]. In addition, a recent report from Guizhou indicated that Cd concentrations in HCT ranged from 76.3 to 226.4 ng/g (dry weight) [41], while HCT from the Beiya area exhibited elevated Cr concentrations [39]. These findings indicate that HCT is capable of accumulating several food-relevant toxic elements, especially under geochemically enriched or mining-impacted conditions.

Although previous studies have provided useful evidence on metal accumulation in HCT, current knowledge remains only partly sufficient for characterizing consumer exposure through market-sold products. Many available investigations have been conducted in specific production areas, experimental systems, or mining-impacted sites, which are important for understanding source-related contamination mechanisms but may not fully represent the HCT actually purchased and consumed through local markets. Market-sold HCT can originate from multiple production areas and pass through complex circulation channels, potentially leading to contamination patterns that differ from those observed in single-source field surveys. In addition, existing studies have commonly focused on one element or a limited set of contaminants, whereas Hg, As, Pb, Cd, and Cr differ in sources, environmental behavior, plant uptake, regulatory significance, and toxicological effects. In a region such as Guizhou, where karst geology, historical mining activities, agricultural production, and market circulation coexist, these elements may show distinct spatial distributions and risk contributions. Therefore, concentration data for individual elements or isolated sampling sites may provide an incomplete basis for risk-oriented management. A more integrated market-based assessment is needed to link multi-element contamination profiles, spatial heterogeneity, and dietary health-risk evaluation. Such an approach can help identify the dominant contaminants and priority risk areas in market-sold HCT, thereby providing more directly relevant evidence for consumer protection and regional food safety management.

To address these knowledge gaps, this study conducted a province-wide point-of-purchase assessment of Hg, As, Cd, Pb, and Cr in HCT rhizomes obtained from retail markets across Guizhou, China. The specific objectives were to: characterize the concentrations and distributional profiles of the five toxic elements in market-level composite samples; evaluate regulatory compliance using food-category-specific maximum limits, including the Cd limit applicable to stem vegetables; examine geographic variability in concentrations across the retail-market network; evaluate multi-element co-occurrence patterns; and estimate screening-level non-carcinogenic dietary exposure under typical and hypothetical high-ingestion scenarios. Because the products were collected at retail markets and their original production locations were not fully traceable, the geographic patterns were interpreted as point-of-purchase patterns rather than as direct representations of contamination in local agricultural soils or production areas.

## 2. Materials and Methods

### 2.1. Study Area and Market-Based Sampling Design

This study was conducted in Guizhou Province, China, which comprises nine prefecture-level administrative divisions and 76 county-level administrative units. Sampling was conducted from April to August 2023, corresponding to the main marketing season of HCT. A total of 101 market-level composite samples were collected across the nine prefecture-level areas of Guizhou Province, comprising 20 samples from Zunyi, 18 from Guiyang, 13 from Tongren, 10 from Anshun, nine each from Bijie and Qiannan, eight from Qiandongnan, and seven each from Liupanshui and Qianxinan (Table S3). The regional sample allocation was broadly determined according to population size, the number and distribution of retail markets, geographic coverage, and field accessibility, with more samples collected from populous areas with larger retail-market networks. Each composite sample comprised HCT purchased from three independent vendors.

HCT rhizomes were purchased from major retail vegetable markets in county seats or principal urban centers. Each market represented one sampling unit. Within each market, HCT was purchased from at least three spatially separated vendors operating as independent retail sellers. Equal fresh-weight portions obtained from the selected vendors were pooled to generate one market-level composite sample of approximately 1.0 kg. Sample-level information, including sampling date, county, prefecture, market type, geographic coordinates, and the number of vendors contributing to each composite sample, is provided in Supplementary Table S3. This composite-sampling strategy was used to characterize the average point-of-purchase concentration at the market level while maintaining province-wide geographic coverage and a manageable analytical workload. Where more than one market was sampled within the same prefectural capital, the markets were selected from different urban areas and each composite sample was retained as a separate point-of-purchase observation. These samples were included individually in the descriptive and spatial analyses using their respective market coordinates. For prefecture-level summaries, all market-level samples within each prefecture were used to calculate the corresponding regional statistics. The selected vendors operated independently and were located at different stalls; however, the original production locations and wholesale-distribution channels of the purchased HCT were not consistently traceable. Therefore, independence at the production-source level could not be confirmed, and the possibility that products from different vendors or markets shared a common wholesale source cannot be completely excluded.

### 2.2. Sample Digestion and Elemental Analysis

#### 2.2.1. Sample Preparation, Moisture Determination, and Fresh-Weight Conversion

After collection, the HCT rhizomes were thoroughly washed with deionized water to remove adhering soil and other surface impurities. The cleaned samples were placed at room temperature until no visible surface water remained and were then weighed immediately to obtain the fresh mass. Subsequently, the samples were oven-dried at 40 °C to constant mass, following low-temperature drying procedures previously applied or validated for total Hg determination in vegetation and other plant matrices [42,43,44,45], cooled to room temperature in a desiccator, and weighed again to obtain the dry mass. Moisture content was calculated as the percentage of mass lost during drying relative to the initial fresh mass.

Moisture content was determined individually for all 101 HCT rhizome samples. As shown in Figure S1, the overall mean moisture content was 76.49 ± 7.40%, with a median of 77.30% and a range of 48.30–91.91%. The corresponding average dry-matter proportion was approximately 23.51% of the fresh mass. Regional mean moisture contents ranged from 69.94% in Bijie to 80.64% in Zunyi. Relatively high mean values were also observed in Anshun (80.52%), Liupanshui (80.28%), and Tongren (78.61%), followed by Qiannan (77.53%) and Qianxinan (77.37%). Comparatively lower mean moisture contents were recorded in Guiyang (71.67%) and Qiandongnan (71.40%). Because moisture content varied considerably among individual samples and regions, the concentration of each element measured on a dry-weight basis was converted to a fresh-weight basis using the dry-to-fresh mass ratio determined separately for the corresponding sample, rather than using the overall mean moisture content. The resulting fresh-weight concentrations were used for comparison with the maximum limits specified in GB 2762–2022 [46], whereas dry-weight concentrations were retained for comparisons with previous studies and for multivariate statistical analyses.

#### 2.2.2. Microwave Digestion for As, Cd, Pb, and Cr

Microwave digestion was performed using a laboratory-established closed-vessel procedure adapted, with modifications, from the plant-matrix digestion method reported by a previous study [47]. Approximately 0.2 g of dried HCT rhizome powder was accurately weighed into polytetrafluoroethylene (PTFE) microwave digestion vessels. Each vessel received 5 mL of ultrapure HNO_3_ and 1 mL of trace-metal-grade H_2_O_2_. After predigestion at room temperature, the samples were digested in an Aurora 6 microwave digestion system (Preekem Scientific Instruments Co., Ltd., Shanghai, China) using a three-step temperature program adapted from published plant-matrix microwave digestion protocols: 120 °C for 6 min, 150 °C for 3 min, and 190 °C for 8 min. After cooling to room temperature, the digests were quantitatively transferred to polypropylene tubes and brought to a final volume of 25 mL with ultrapure water. Reagent blanks were prepared and processed in parallel using the same digestion and dilution procedures.

#### 2.2.3. Determination of Hg, As, Cd, Pb and Cr

Total Hg was measured using a direct mercury analyzer (DMA-80, Milestone, Sorisole, Italy) following established procedures [45,48]. Dried HCT rhizome powders were analyzed directly without wet digestion. The samples underwent thermal decomposition, catalytic conversion, gold amalgamation preconcentration, and thermal desorption, and atomic absorption detection at 253.7 nm. Total As was quantified using hydride generation atomic fluorescence spectrometry (HG-AFS) following an established method for plant matrices [49]. After the conventional pre-reduction step, As was reduced with potassium borohydride (KBH_4_) to generate volatile hydrides, which were transported by an inert carrier gas into the atomizer. The fluorescence signal was measured at the analytical wavelength and quantified using external calibration. Cd, Pb, and Cr were determined by inductively coupled plasma mass spectrometry (ICP-MS; iCAP RQ, Thermo Fisher Scientific, Waltham, MA, USA) following established procedures for plant and food matrices [50]. The monitored isotopes were ^111^Cd, ^208^Pb, and ^52^Cr for Cd, Pb, and Cr, respectively. An internal-standard mixture containing Sc, In, and Tl was introduced online to correct for instrumental drift and matrix effects. Measurements were performed in helium kinetic-energy-discrimination mode to suppress polyatomic interferences. The instruments were operated using their standard analytical programs, and the principal operating conditions were optimized before each analytical sequence. The method detection limits (LODs) and limits of quantification (LOQs) were 0.173 and 0.577 ng/g DW for Hg, 1.1 and 3.7 ng/g DW for As, 0.187 and 0.623 ng/g DW for Cd, 0.325 and 1.08 ng/g DW for Pb, and 0.218 and 0.727 ng/g DW for Cr, respectively. Only As included measurements below the LOD. For statistical analysis, As concentrations below the LOD were replaced with one-half of the LOD (0.55 ng/g DW), whereas measurements at or above the LOD were retained. The minimum concentrations of Cd, Pb, Cr, and Hg were all above their corresponding LOQs; therefore, no substitution of no detects was required for these elements.

### 2.3. Quality Assurance and Quality Control (QA/QC)

Calibration standards were prepared from certified stock solutions, and all calibration curves showed satisfactory linearity (R^2^ > 0.999). Each analytical batch, comprising approximately 20 samples, included procedural blanks, duplicate samples (≥10% of the samples), and the plant certified reference materials (CRMs) GBW 10020 (Citrus leaf) and GBW(E) 090066 (*Salvia miltiorrhiza*). Method accuracy was evaluated using these two CRMs. The certified concentrations of Hg, As, Cd, Cr, and Pb in GBW 10020 were 0.150 ± 0.020, 1.10 ± 0.20, 0.170 ± 0.020, 1.25 ± 0.11, and 9.70 ± 0.90 mg/kg DW, respectively. The certified concentrations of Hg, As, Cd, and Pb in GBW(E) 090066 were 0.012 ± 0.001, 0.40 ± 0.06, 0.047 ± 0.004, and 0.93 ± 0.04 mg/kg DW, respectively; no certified value was provided for Cr in this reference material. The CRMs were processed in parallel with the samples. For As, Cd, Pb, and Cr, appropriate aliquots of the CRMs were subjected to the same sample mass, acid composition, microwave digestion program, final dilution volume, and instrumental determination as the HCT samples. Reagent blanks containing the same quantities of HNO_3_ and H_2_O_2_ but no sample material were carried through the complete digestion procedure. For Hg, aliquots of the CRMs were analyzed directly using the same DMA-80 program as the HCT samples, and empty sample boats were included as analytical blanks. The recovery ranges calculated from the CRM measurements across the analytical batches were 89.7–107.3% for Hg, 86.7–112.5% for As, 90.3–104.6% for Cd, 87.3–107.6% for Cr, and 88.3–97.8% for Pb. All recoveries were within the acceptance range of 85–115%, and the relative standard deviations of duplicate measurements were below 10%. All containers and sample-handling tools were acid-cleaned and rinsed thoroughly with ultrapure water before use to minimize contamination.

### 2.4. Spatial Visualization of Point-of-Purchase Patterns

Spatial interpolation was performed in ArcGIS Desktop 10.3 (Esri, Redlands, CA, USA). Element concentrations were natural-log-transformed before analysis. Simple Kriging was used for Cd, As, Cr, and Hg, and Ordinary Kriging was used for Pb; exponential models were fitted for Cd and Cr, and stable models for Pb, As, and Hg. Model performance was evaluated by leave-one-out cross-validation using mean error (ME) as a measure of bias and MAE and RMSE as measures of prediction error. Kriging was also compared with IDW using the same sampling locations and validation procedure. The model parameters and validation results are provided in Table S2. The interpolated surfaces were used only to visualize point-of-purchase variation and not to infer production-source contamination.

### 2.5. Integrated Health-Risk Assessment

Non-carcinogenic health risks associated with dietary exposure to Hg, As, Cd, Pb, and Cr through the consumption of HCT were assessed using an integrated estimated daily intake (EDI), target hazard quotient (THQ), and hazard index (HI) framework. The general exposure model followed the approach recommended by the U.S. Environmental Protection Agency (USEPA) [51,52], while element-specific oral reference values were selected by integrating current USEPA reference doses (RfDs) and health-based guidance values established by the Joint FAO/WHO Expert Committee on Food Additives (JECFA). When both organizations provided applicable values, the lower daily-equivalent value was selected to obtain a conservative screening estimate.

The EDI for each element was calculated as:

(1) $$EDI_{i}\;=\;\frac{C_{i}\;\times \;IR\;\times \;EF\;\times \;ED}{BW\;\times \;AT}$$

where EDI_i_ is the estimated daily intake of element i, expressed as μg/(kg·bw·day); C_i_ is the concentration of element i in HCT rhizomes (μg/kg), fresh weight); IR is the daily ingestion rate of HCT (g/day); EF is the exposure frequency; ED is the exposure duration (years); BW is body weight (kg); and AT is the averaging time for non-carcinogenic effects. For the non-carcinogenic assessment, EF was set at 365 days/year and AT was defined as ED × 365 days. Therefore, EF, ED, and AT cancel from the general equation:

(2) $$EDI_{i}\;=\;\frac{C_{i}\;\times \;IR\;\times \;10^{-3}}{BW}$$

The calculated EDI therefore represents the long-term daily-equivalent exposure attributable to HCT consumption.

The dietary-exposure assessment was restricted to adults. A representative adult body weight of 60 kg was used for all three ingestion scenarios. The low-ingestion scenario was set at 30 g/day, representing light but regular consumption. The medium-ingestion scenario was set at 76 g/day, based on the previously reported local dietary intake of HCT in Guizhou [27]. The high-ingestion scenario was set at 150 g/day, approximately twice the locally reported mean intake and representative of a large HCT serving.

To integrate the USEPA and JECFA health-based guidance values within a common THQ framework, the element-specific daily oral reference values (RV_i_) were uniformly expressed as μg/(kg·bw·day). The selected RV_i_ values were 0.571 μg/(kg·bw·day) for Hg, 0.06 μg/(kg·bw·day) for As, 0.82 μg/(kg·bw·day) for Cd, 3.6 μg/(kg·bw·day) for Pb, and 0.90 μg/(kg·bw·day) for Cr [53,54,55,56,57,58,59,60]. For Hg, total Hg was evaluated as inorganic Hg using a daily-equivalent reference value of 0.571 μg/(kg bw·day), derived from the JECFA PTWI of 4 μg/(kg bw·week) for inorganic Hg [56]. For As, total As was conservatively treated as inorganic As. Non-carcinogenic risk was evaluated using the updated USEPA IRIS oral reference dose of 0.060 μg/(kg bw·day), derived on the basis of ischemic heart disease and type 2 diabetes in humans [61]. Because only total As was measured, all detected As was conservatively assumed to be inorganic As. For Cd, the JECFA PTMI of 25 μg/(kg·bw·month) [57], yielding a daily-equivalent $RV_{i}$ of 0.82 μg/(kg·bw·day), which is lower than the USEPA dietary Cd RfD of 1.0 μg/(kg·bw·day) [58]. For Pb, the USEPA has not established an oral RfD, and JECFA has withdrawn its former PTWI of 25 μg/(kg·bw·week) [56,60]. The former PTWI was withdrawn because exposure at that level was associated with an estimated decrease of at least three IQ points in children and an increase of approximately 3 mmHg in adult systolic blood pressure, and no threshold was identified for these critical effects. Nevertheless, to retain Pb in the screening-level THQ and HI calculations and maintain comparability with earlier food-contamination studies, the daily-equivalent value of 3.6 μg/(kg·bw·day), derived from the former PTWI, was retained as a historical screening reference value. This value was not interpreted as a current health-protective threshold, and a Pb-THQ below 1 was not considered evidence of an absence of Pb-related health risk. For Cr, the current USEPA oral RfD of 0.90 μg/(kg·bw·day) for Cr(VI) was used [59]. Because only total Cr was measured, all measured Cr was conservatively assumed to occur as Cr(VI). Consequently, the As- and Cr-related risk estimates were interpreted as upper-bound screening estimates.

The THQ for each element was calculated as:

(3) $$THQ_{i}\;=\;\frac{EDI_{i}}{RV_{i}}$$

where RV_i_ is the selected element-specific daily oral reference value. The cumulative non-carcinogenic screening risk was expressed as:

(4) $$HI\;=\;\sum_{i=1}^{5}THQ_{i}$$

where the five terms represent Hg, As, Cd, Pb, and Cr.

For Hg, As, Cd, and Cr, a THQ below 1 indicates that estimated exposure was below the selected health-based reference value under the stated exposure and speciation assumptions, whereas a THQ equal to or above 1 indicates a need for further refined assessment. An HI below 1 indicates that the combined exposure estimate was below the summed screening benchmarks, while an HI equal to or above 1 indicates potential cumulative concern. However, because total As and total Cr were conservatively treated as inorganic As and Cr(VI), respectively, and because Pb was evaluated using a withdrawn historical JECFA value, the calculated HI was interpreted as a comparative screening index with element-specific toxicological limitations rather than a quantitative prediction of adverse health effects. To support the integrated multi-element assessment, the total-Hg data for the market-level composite samples were derived in part from the same underlying retail samples examined in a related Hg–Se study currently under review [62]; that study focused specifically on Hg–Se co-exposure and selenium-based health-benefit indices.

Because no health-protective exposure threshold has been identified for Pb, the screening-level HI assessment was supplemented by an effect-based MOE analysis. Two adult reference points established by the European Food Safety Authority were applied [63,64]. The renal reference point was the benchmark dose lower confidence limit for a 10% response (BMDL10) of 0.63 μg/(kg bw·day), based on an increased prevalence of chronic kidney disease. The cardiovascular reference point was the benchmark dose lower confidence limit for a 1% response (BMDL01) of 1.50 μg/(kg bw·day), based on an increase in systolic blood pressure [63,64]. The renal endpoint was treated as the primary endpoint because it had the lower and therefore more sensitive reference point, while the cardiovascular endpoint was used as a complementary assessment. The Pb MOEs were calculated as:

(5) $${MOE}_{\mathrm{Pb},\mathrm{renal}}\;=\;\frac{{BMDL}_{10,\mathrm{renal}}}{EDI_{\mathrm{Pb}}}\;=\;\frac{0.63}{{EDI}_{\mathrm{Pb}}}$$

(6) $${MOE}_{\mathrm{Pb},\mathrm{cardiovascular}}\;=\;\frac{{BMDL}_{01,\mathrm{cardiovascular}}}{{EDI}_{\mathrm{Pb}}}\;=\;\frac{1.50}{{EDI}_{\mathrm{Pb}}}$$

where EDI_Pb_ is the estimated daily intake of Pb under the corresponding consumption scenario. A larger MOE indicates a greater separation between the estimated Pb exposure and the corresponding effect-based reference point. For screening and risk prioritization, MOE values were classified into three categories: MOE ≥ 10, for which an appreciable risk of a clinically significant effect is unlikely; MOE from 1 to <10, for which the possibility of an effect cannot be excluded; and MOE < 1, indicating that the estimated Pb exposure reaches or exceeds the corresponding effect-based reference point and therefore warrants further investigation. These categories were used for risk prioritization and were not interpreted as strict boundaries between safe and unsafe exposure.

Both the comparative HI and Pb MOE assessments were based on measured fresh-weight concentrations and assumed continuous long-term consumption. No reductions arising from washing, cooking, or other food-preparation processes were considered, and gastrointestinal bioaccessibility and element-specific absorption were not incorporated. Furthermore, the Pb MOEs represented exposure attributable to HCT alone and did not include Pb intake from other foods, drinking water, air, soil, or dust. The results therefore characterize the incremental contribution of HCT consumption rather than total dietary and environmental exposure.

### 2.6. Statistical Analysis and Multi-Element Co-Occurrence Assessment

The distributions of Hg, As, Cd, Pb, and Cr concentrations were assessed using the Shapiro–Wilk test together with visual inspection of histograms and quantile–quantile plots. Because several element distributions, particularly those of Hg, As, and Pb, were strongly right-skewed and contained extreme values, pairwise associations among the five elements were evaluated using Spearman’s rank correlation coefficient (ρ). A total of 10 pairwise correlation tests were conducted. To account for multiple comparisons, the raw *p* values were adjusted using the Benjamini–Hochberg false discovery rate procedure, and an FDR-adjusted *p* value (q) of less than 0.05 was considered statistically significant. The correlation coefficients were interpreted conservatively as measures of monotonic co-occurrence at the point of purchase and were not treated as direct evidence of common contamination sources.

Principal component analysis (PCA) was performed on standardized concentration data to summarize the major compositional gradients among the five elements. Components with eigenvalues greater than 1 were retained. PCA loadings and scores were interpreted as descriptive multivariate patterns rather than definitive source categories. Statistical analyses were performed using SPSS version 26.0, while spatial maps and other figures were prepared using ArcGIS 10.8 and standard scientific plotting software.

## 3. Results and Discussion

### 3.1. Occurrence of Hg, As, Cd, Pb and Cr in Retail HCT

Figure 1 shows the occurrence of Hg, As, Cd, Pb, and Cr in retail HCT rhizomes collected across Guizhou Province. On a fresh-weight (FW) basis, the mean ± SD concentrations (ng/g) were 4.8 ± 8.7 for Hg, 4.1 ± 20.8 for As, 111.5 ± 64.6 for Cd, 291.3 ± 295.0 for Pb, and 6.3 ± 3.7 for Cr. The corresponding medians were 3.4, 0.5, 99.8, 184.3, and 5.4 ng/g, respectively, and the concentration ranges were 0.8–79.5 for Hg, 0.0–203.5 for As, 19.8–297.1 for Cd, 18.3–1328.0 for Pb, and 2.1–18.9 ng/g for Cr. On a dry-weight (DW) basis, the same overall pattern was retained, with mean concentrations of 20.4 ± 24.2, 15.1 ± 62.8, 503.3 ± 254.1, 1287.2 ± 1257.3, and 27.3 ± 11.3 ng/g for Hg, As, Cd, Pb, and Cr, respectively. Pb and Cd occurred at substantially higher mean concentrations than Hg, As, and Cr in the market-level composite samples.

The distributional characteristics differed markedly among the five elements. Hg had a relatively low median concentration of 3.4 ng/g (FW) but a maximum concentration of 79.5 ng/g (FW), indicating that most market-level composite samples contained low Hg concentrations, while a small number of samples contributed disproportionately to the upper tail of the distribution. A similar but more strongly right-skewed pattern was observed for As: the median concentration was only 0.5 ng/g (FW), whereas the maximum reached 203.5 ng/g (FW). This contrast indicates that As was generally present at low concentrations across the sampled retail markets but was occasionally elevated in individual market-level samples. In comparison, Cd showed a narrower and more consistently elevated distribution, with concentrations ranging from 19.8 to 297.1 ng/g (FW). Its median concentration of 99.8 ng/g (FW) was close to the mean and nearly identical to the maximum limit applicable to stem vegetables, indicating that the point-of-purchase Cd concentrations were broadly distributed around the regulatory threshold rather than being driven primarily by a small number of extreme values. Pb combined a high central tendency with pronounced variability, with a median concentration of 184.3 ng/g (FW) and a maximum of 1328.0 ng/g (FW), indicating both widespread elevation and substantial variation among the sampled retail markets. Cr showed the lowest variability among the five elements, with a relatively narrow concentration range and a mean only slightly higher than the median, reflecting a comparatively stable and low-level occurrence pattern.

Comparison with the maximum limits specified in the National Food Safety Standard—Maximum Levels of Contaminants in Foods (GB 2762–2022) [46] further demonstrated marked differences in regulatory compliance among the five elements. Because the edible rhizome of HCT is botanically a modified underground stem, it was classified as a stem vegetable for regulatory comparison. Mean Hg remained below the maximum limit of 10 ng/g (FW), and only 4 of the 101 market-level composite samples (4.0%) exceeded this threshold. Mean As was substantially below its maximum limit of 500 ng/g (FW), and no As exceedances were observed despite the strongly right-skewed concentration distribution. These findings indicate that Hg and As made relatively limited contributions to overall regulatory non-compliance across the sampled retail markets. The mean Cd concentration was 111.5 ng/g (FW), approximately 1.12 times the maximum limit for stem vegetables. A total of 50 of the 101 market-level composite samples exceeded the Cd limit, corresponding to an exceedance frequency of 49.5%. The median Cd concentration was 99.8 ng/g (FW) and was therefore close to, but slightly below, the applicable maximum limit. These results indicate that the point-of-purchase Cd distribution was centered near the regulatory threshold, with approximately half of the sampled market composites exceeding the limit. Pb showed more extensive regulatory non-compliance. Its mean concentration was 291.3 ng/g (FW), approximately 2.9 times the maximum limit of 100 ng/g (FW), and 88 of the 101 samples (87.1%) exceeded the standard. The median Pb concentration of 184.3 ng/g (FW) also substantially exceeded the corresponding limit, indicating that the high Pb exceedance frequency was not attributable solely to a small number of extreme samples. Cr remained below its maximum limit of 500 ng/g (FW) in all samples. Overall, Pb represented the element with the most widespread regulatory exceedance, whereas Cd remained an important compliance concern because its mean concentration exceeded the applicable limit and approximately half of the market-level composite samples were non-compliant.

The point-of-purchase patterns observed in retail HCT should be interpreted within the broader environmental and commercial context of Guizhou. Previous province-scale investigations [31] have shown that agricultural soils in Guizhou can be influenced by naturally elevated geochemical backgrounds, historical mining and smelting activities, industrial emissions, and agricultural inputs. However, because the HCT samples in the present study were obtained from retail markets and their original production locations and supply chains were not fully traceable, the measured concentrations cannot be directly attributed to environmental conditions in the counties where the products were purchased. The relatively low mean Hg concentration in the retail samples, compared with the much higher concentrations previously reported in crops from heavily Hg-impacted mining areas [27,37], suggests that products originating from severely contaminated Hg-mining areas did not dominate the overall retail sample set. Nevertheless, the small number of samples with elevated Hg concentrations indicates that specific supply chains may warrant further tracing. For Cd, the mean concentration exceeded the maximum limit for stem vegetables, and approximately half of the market-level composite samples were non-compliant. This pattern supports the need for broad market surveillance but does not, by itself, distinguish among contributions from geochemical background, agricultural practices, industrial activities, or product circulation. Pb showed the highest median concentration and the most extensive regulatory exceedance, making it the primary point-of-purchase compliance concern among the five elements. As was generally low but occasionally elevated in individual samples, whereas Cr remained comparatively low and stable. Overall, the retail-market evidence revealed distinct element-specific point-of-purchase profiles: Hg and As were characterized by predominantly low concentrations with occasional upper-tail values; Cd concentrations were broadly distributed around the regulatory threshold for stem vegetables; Pb showed widespread and pronounced regulatory non-compliance; and Cr remained consistently low. These findings support strengthened retail-market monitoring and supply-chain tracing, rather than direct attribution of the observed concentrations to local production environments or specific pollution sources.

### 3.2. Kriging-Interpolated Point-of-Purchase Patterns in Guizhou Retail Markets

Figure 2 presents the Kriging-interpolated point-of-purchase patterns of the five elements across Guizhou retail markets. The Kriging models showed acceptable leave-one-out cross-validation performance (Table S4), with mean standardized errors ranging from −0.0142 to 0.0730 and RMS standardized errors ranging from 0.9047 to 1.1425. Kriging produced lower MAEs and RMSEs than IDW for all five elements, reducing RMSE by approximately 7.3–8.7% on the natural-log-transformed scale. The nugget-to-sill ratios indicated strong spatial dependence among the sampled retail locations for As, Cr, Cd, and Pb and moderate spatial dependence for Hg. These results supported the use of Kriging for exploratory visualization of market-level variation.

As shown in Figure 2, Hg and As concentrations were generally low across most sampled markets, although relatively elevated values occurred in several locations. Higher Hg values were observed mainly in Guanling and parts of Tongren, whereas relatively high As values occurred in Guanling, Huangping, and Suiyang. These observations identify retail supply chains that may warrant repeated sampling and origin tracing but do not permit attribution to local environmental sources. Cd and Pb showed more extensive distributions of elevated point-of-purchase values. Cd concentrations near or above the regulatory limit for stem vegetables occurred in markets in Tongren, Zunyi, Bijie, and Qiannan. Pb showed the most widespread regulatory non-compliance, with relatively high interpolated values in Tongren, Qianxinan, southern Guizhou, and parts of Bijie, consistent with its 87.1% exceedance frequency. Cr varied among retail locations, with relatively high values in Bijie, Tongren, Qiandongnan, and Zunyi, although all measured concentrations remained below the regulatory limit.

Hg and As elevations were restricted to a small number of markets, Cd concentrations were broadly distributed around the regulatory threshold, Pb showed widespread non-compliance, and Cr remained comparatively low. These findings support repeated sampling and supply-chain tracing for elevated Hg and As observations and broader retail surveillance for Cd and Pb. Because shared wholesale sources may connect distant markets and neighboring markets may obtain HCT from unrelated production areas, the interpolated surfaces should not be interpreted as production-source contamination or spatial hotspots. Markets were sampled on different dates between April and August 2023 without repeated seasonal sampling; the patterns therefore represent cross-sectional point-of-purchase variation during the survey period rather than stable spatial or seasonal distributions.

### 3.3. Multi-Element Co-Occurrence and Potential Source Signals

Spearman’s rank correlation analysis was used because the concentration distributions of all five elements deviated significantly from normality (Table S1), even after log_10_ transformation. After adjustment for 10 pairwise comparisons using the Benjamini–Hochberg false discovery rate procedure, four weak positive associations remained statistically significant (Figure 3, Table S2). These included Hg–As (ρ = 0.254, q = 0.026), Hg–Pb (ρ = 0.272, q = 0.019), As–Pb (ρ = 0.290, q = 0.016), and Cd–Pb (ρ = 0.323, q = 0.0098). The nominal association between Hg and Cd (ρ = 0.205, raw *p* = 0.040) did not remain significant after FDR correction (q = 0.079). No significant associations were detected between Cr and any of the other four elements.

Although four element pairs remained significant after multiple-comparison correction, all coefficients were weak, with the largest value being only 0.323. The results therefore indicate limited pairwise co-occurrence rather than strong or uniform multi-element enrichment across the retail-market samples. Pb showed weak positive associations with Hg, As, and Cd, but these relationships do not demonstrate a common environmental source. Because the samples were collected at retail markets and their production origins and distribution pathways were not fully traceable, the observed associations may reflect a combination of mixed production origins, commodity circulation, environmental exposure, and plant-uptake behavior. Consequently, the correlation results should be interpreted as descriptive point-of-purchase co-occurrence patterns and cannot independently support source attribution.

Principal component analysis (PCA) was performed using log_10_-transformed and standardized dry-weight concentrations from the 101 market-level composite samples (Figure 4). The KMO value was 0.641, and Bartlett’s test of sphericity was significant (χ^2^ = 50.880, df = 10, *p* < 0.001), indicating a moderate but sufficient correlation structure for exploratory dimension reduction. PC1 and PC2 explained 38.21% and 21.14% of the total variance, respectively, yielding a cumulative explained variance of 59.35%. Although both components had eigenvalues greater than 1, Horn’s parallel analysis provided clear support only for PC1. PC2 was therefore retained mainly for two-dimensional visualization and was interpreted cautiously.

PC1 showed high positive loadings for Pb (0.732), Hg (0.731), and As (0.667), together with a moderate positive loading for Cd (0.517), whereas the loading of Cr was comparatively low (0.358). Pb, Hg, and As contributed 28.07%, 27.97%, and 23.26% to PC1, respectively. PC1 therefore represented a general multi-element concentration gradient rather than a source-specific Hg–As factor. Samples with high PC1 scores tended to contain jointly elevated Hg, As, Pb, and, to a lesser extent, Cd. This interpretation was broadly consistent with the weak positive Spearman associations observed among Hg, As, Pb, and Cd. Nevertheless, the moderate loadings and weak pairwise correlations do not support the existence of a single common contamination source. PC2 was characterized mainly by positive loadings for Cr (0.690) and Cd (0.583), which together accounted for 77.23% of the component contribution, while As showed a negative loading of −0.409. PC2 was therefore interpreted as a secondary Cd–Cr-oriented compositional gradient. This axis should not be regarded as evidence that Cd and Cr originated from a common source, because their pairwise correlation was weak and non-significant. Rather, PC2 described differences in relative elemental profiles after the general concentration gradient represented by PC1 had been removed.

The score plot showed substantial overlap among the nine prefecture-level regions. Tongren had the highest mean PC1 score, whereas Guiyang had the lowest, while Bijie showed the highest mean PC2 score. However, within-region variability was considerable, and individual extreme samples strongly affected the apparent position and dispersion of some groups, particularly Anshun. Moreover, because the samples were obtained from retail markets and their production origins were not fully traceable, the score patterns represent point-of-purchase compositional variation rather than local environmental source signatures. PCA should therefore be considered an exploratory summary of multi-element co-variation and not a source-apportionment model.

### 3.4. Dietary Exposure and Screening-Level Health-Risk Assessment

Dietary exposure was evaluated under the low-, medium-, and high-consumption scenarios. The five-element HI incorporated Hg, As, Cd, Pb, and Cr and was used as an integrated screening indicator of the combined element burden. Pb was included in this index using the historical daily-equivalent value described in Section 2.5, thereby permitting comparison of its relative contribution with those of the other elements. Accordingly, the HI was interpreted as a screening-level indicator rather than as a quantitative prediction of cumulative adverse effects. The overall HI distribution is presented first, followed by prefecture-level variation and the supplementary effect-based assessment of Pb using the MOE approach.

#### 3.4.1. Overall Distribution of the Five-Element Comparative Screening Hazard Index (HI)

The distribution of the five-element comparative screening hazard index HI shifted progressively upward with increasing HCT consumption (Table 1). Under the low-consumption scenario, the mean HI was 0.235, with a median of 0.200 and an interquartile range of 0.137–0.270. Under the medium-consumption scenario, the mean HI increased to 0.381, while the median was 0.274 and the interquartile range was 0.179–0.448. Under the high-consumption scenario, the mean and median HIs reached 0.751 and 0.542, respectively, with an interquartile range of 0.353–0.883. Thus, both the mean and median remained below 1 under all three scenarios, although the central tendency moved closer to 1 as the assumed consumption level increased.

The distributions were right-skewed, particularly under the medium- and high-consumption scenarios. The maximum HI values were 2.017, 4.958, and 9.786 under the low-, medium-, and high-consumption scenarios, respectively, and were substantially higher than the corresponding medians. The 95th-percentile HI increased from 0.433 under the low-consumption scenario to 0.793 under the medium-consumption scenario and 1.566 under the high-consumption scenario. The high-consumption P95 exceeded 1, indicating that the upper end of the sample distribution reached a comparatively elevated combined element burden even though the overall mean and median remained below 1.

Among the 101 retail-market composite samples, 1.0% had an HI above 1 under the low-consumption scenario. This proportion increased to 3.0% under the medium-consumption scenario and 16.8% under the high-consumption scenario. Therefore, most samples remained below the screening threshold under the low- and medium-consumption assumptions, whereas high consumption substantially increased the proportion of samples with elevated HIs. The mean and median alone consequently provide an incomplete representation of the exposure distribution, and upper-percentile values and the frequency of HI exceedance are also important for identifying retail samples warranting further attention.

Because the HI was intended as a screening-level index, an HI above 1 identifies a comparatively elevated combined element burden requiring further evaluation but does not directly predict that adverse health effects will occur. Conversely, an HI below 1 should not be interpreted as definitive evidence of an absence of risk, particularly because Pb was incorporated using a historical normalization benchmark rather than a current health-protective threshold. The supplementary Pb MOE assessment presented in Section 3.4.4 provides a more effect-based characterization of Pb-related concern.

#### 3.4.2. Element-Specific Contributions to the Five-Element Comparative Screening HI

To identify the elements driving the five-element comparative screening HI described above, the element-specific quotients and their relative contributions were further examined (Table 2). Because all element-specific quotients increased proportionally with the assumed ingestion rate, their percentage contributions to the HI remained unchanged across the low-, medium-, and high-consumption scenarios. Contributions were first calculated separately for each sample as THQ_i_/HI× 100% and were then summarized across the 101 market-level composite samples.

Cd was the predominant component of the five-element HI. Its median quotient increased from 0.0603 under low consumption to 0.1527 under medium consumption and 0.3013 under high consumption. Cd accounted for a mean of 55.5% of the sample-level HI, with a median contribution of 59.4% (IQR: 47.6–69.8%). Although Cd was the largest contributor in most samples, none of the individual Cd quotients exceeded 1 under any consumption scenario.

Pb was the second-largest contributor, accounting for a mean of 29.0% of the HI. Its median contribution was 25.3% (IQR: 19.5–35.0%). The median Pb quotient increased from 0.0250 to 0.0634 and 0.1251 across the low-, medium-, and high-consumption scenarios, respectively. No Pb quotient exceeded 1 under low or medium consumption, whereas one sample (1.0%) exceeded 1 under high consumption. Together, Cd and Pb accounted for an average of 84.5% of the sample-level HI, demonstrating that these two elements dominated the integrated screening index. As noted above, however, the Pb component was based on a historical normalization benchmark and should be interpreted only within the comparative screening framework.

As generally made a smaller contribution than Cd and Pb, but its distribution showed considerably greater between-sample variability. The median As contribution was 4.0% (IQR: 2.7–6.7%), whereas its mean contribution was 9.1%. Its 95th-percentile contribution reached 49.5%, indicating that As became a major component of the HI in a small number of samples. Correspondingly, the median As quotients were only 0.0042, 0.0101, and 0.0191 under the three consumption scenarios, but the respective maximum values reached 1.617, 4.096, and 8.085. The number of samples with an As quotient above 1 increased from one (1.0%) under low consumption to two (2.0%) under medium consumption and three (3.0%) under high consumption. These analytically verified upper-tail observations were retained because they represent potentially important market-level exposure rather than statistical errors.

Hg and Cr contributed relatively little to the five-element HI. Their mean sample-level contributions were both approximately 3.2%, and their median contributions were 2.8% each. Neither Hg nor Cr had an element-specific quotient above 1 under any consumption scenario. Overall, the typical contribution pattern was Cd > Pb > As > Hg ≈ Cr, although the contribution of As increased markedly in a small subset of samples.

These findings show that the provincial HI distribution was driven mainly by the consistently large contributions of Cd and Pb, whereas unusually high As concentrations influenced the upper tail in a limited number of markets. The following section therefore examines whether the magnitude and composition of the five-element HI differed among prefecture-level cities.

#### 3.4.3. Prefecture-Level Variation in the Five-Element Hazard Index

The five-element HI varied among the nine prefecture-level areas and across the three consumption scenarios (Figure 5). Under the low-consumption scenario, the mean HIs were below 1 in all areas. Anshun had the highest mean HI (0.381), followed by Qianxinan (0.375), Bijie (0.309), Qiandongnan (0.297), Qiannan (0.284), and Tongren (0.251). Lower mean values were observed in Liupanshui (0.198), Zunyi (0.149), and Guiyang (0.106). Only one sample had an HI above 1 under this scenario, occurring in Anshun and accounting for 1 of its 10 samples (10.0%). The exceptionally high maximum HI in Anshun (2.017), together with its substantially lower median of 0.200, indicates that the prefecture-level mean was strongly influenced by a single high-value retail-market sample.

Under the medium-consumption scenario, the mean HIs remained below 1 in all nine areas. Anshun again had the highest mean value (0.636), followed by Qianxinan (0.529), Qiandongnan (0.520), Bijie (0.482), and Tongren (0.448). The mean HIs in Qiannan, Zunyi, Guiyang, and Liupanshui ranged from 0.230 to 0.334. Three samples had HIs above 1: one in Anshun (1/10, 10.0%), one in Tongren (1/13, 7.7%), and one in Qiandongnan (1/8, 12.5%). The maximum HI in Anshun reached 4.958, whereas its median was only 0.156, again demonstrating that its elevated mean reflected an isolated extreme value rather than a generally high HI distribution across the sampled markets.

Under the high-consumption scenario, the mean HIs exceeded 1 in Anshun (1.256), Qianxinan (1.044), and Qiandongnan (1.026). The mean HI in Bijie approached 1 (0.951), followed by Tongren (0.885), Qiannan (0.659), Zunyi (0.513), Guiyang (0.467), and Liupanshui (0.455). A total of 17 samples had HIs above 1. These included five samples in Qianxinan (5/7, 71.4%), five in Bijie (5/9, 55.6%), three in Tongren (3/13, 23.1%), and one sample each in Zunyi (1/20, 5.0%), Qiandongnan (1/8, 12.5%), Anshun (1/10, 10.0%), and Qiannan (1/9, 11.1%). No sample from Guiyang or Liupanshui had an HI above 1 under any of the three scenarios.

The prefecture-level means and exceedance frequencies revealed two different distribution patterns. The comparatively high mean HIs in Anshun were driven principally by one extreme retail-market sample, as reflected by the large difference between its mean and median and by the same single exceedance across all three scenarios. Qiandongnan also contained an isolated high-value sample, which increased its mean HI. In contrast, the elevated high-consumption HIs in Qianxinan and Bijie were distributed across a larger proportion of the sampled markets, with five exceedances in each area. Tongren showed an intermediate pattern, with three high-consumption exceedances. Thus, an elevated prefecture-level mean may represent either an isolated extreme market or a more broadly distributed upper-tail burden, and the mean HI should be interpreted together with the number and proportion of samples exceeding 1.

These prefecture-level differences represent point-of-purchase exposure patterns rather than contamination at the place of production. HCT sold in neighboring markets may originate from different production areas, whereas geographically distant markets may share the same wholesale or distribution sources. The results should therefore be interpreted as differences among the sampled retail markets and the consumer populations they serve, rather than as evidence of local soil contamination or geographically defined production sources.

#### 3.4.4. Effect-Based Assessment of Pb Using Margins of Exposure

The Pb MOE results showed a clear consumption-dependent decrease in the separation between estimated exposure and the renal and cardiovascular reference points (Table 3). The renal endpoint consistently produced lower MOEs than the cardiovascular endpoint, indicating that it provided the more sensitive characterization of Pb exposure from HCT consumption.

For the renal endpoint, the median MOE decreased from 6.993 under the low-consumption scenario to 2.760 under the medium-consumption scenario and 1.399 under the high-consumption scenario. Under the low-consumption scenario, 22.8% of the samples had renal MOEs ≥ 10, whereas 76.2% were within the range of 1 to <10 and 1.0% were below 1. Under the medium-consumption scenario, only 1.0% of the samples retained a renal MOE ≥ 10, while 16.8% had MOEs below 1. Under the high-consumption scenario, no sample had a renal MOE ≥ 10, and the proportion with MOE < 1 increased to 32.7%. These results indicate that the margin between HCT-derived Pb exposure and the renal reference point narrowed markedly as the assumed consumption level increased.

Prefecture-level patterns further demonstrated variation among the sampled retail markets. Under the low-consumption scenario, a renal MOE below 1 occurred only in Tongren, accounting for 7.7% of the samples collected in that area. Under the medium-consumption scenario, renal MOEs below 1 occurred in all prefecture-level areas except Zunyi. The highest proportions were observed in Tongren (46.2%), Qianxinan (28.6%), and Qiandongnan (25.0%), followed by Bijie and Qiannan (22.2% each), Liupanshui (14.3%), Anshun (10.0%), and Guiyang (5.6%). Under the high-consumption scenario, renal MOEs below 1 occurred in all nine areas. Qianxinan had the highest proportion (71.4%), followed by Tongren (53.8%) and Qiandongnan (50.0%). The corresponding proportions in the remaining areas ranged from 10.0% in Anshun to 33.3% in both Bijie and Qiannan.

For the cardiovascular endpoint, the median MOEs were 16.650, 6.572, and 3.330 under the low-, medium-, and high-consumption scenarios, respectively. Under the low-consumption scenario, no sample had a cardiovascular MOE below 1, and 74.3% had MOEs ≥ 10. Under the medium-consumption scenario, the proportion with MOE ≥ 10 decreased to 19.8%, while 4.0% of the samples had MOEs below 1. Under the high-consumption scenario, only 4.0% of the samples retained cardiovascular MOEs ≥ 10, whereas 12.9% had MOEs below 1 and 83.2% were within the range of 1 to <10.

No cardiovascular MOE below 1 was observed in any prefecture-level area under the low-consumption scenario. Under the medium-consumption scenario, values below 1 occurred in Tongren (15.4%), Bijie (11.1%), and Anshun (10.0%). Under the high-consumption scenario, cardiovascular MOEs below 1 occurred in seven of the nine areas, with the highest proportions in Tongren (46.2%) and Qianxinan (28.6%). Lower proportions were observed in Liupanshui (14.3%), Qiandongnan (12.5%), Bijie (11.1%), Anshun (10.0%), and Guiyang (5.6%). No cardiovascular MOE below 1 was observed in Zunyi or Qiannan under this scenario.

The results from both endpoints consistently demonstrated that increasing HCT consumption progressively reduced the margin between Pb exposure and the corresponding effect-based reference points. The renal endpoint identified a greater proportion of potentially concerning samples, particularly under the medium- and high-consumption scenarios. The prefecture-level results also indicated that Pb-related concern was not distributed uniformly across the sampled retail system. Tongren and Qianxinan showed comparatively high proportions of low MOEs, although the pattern varied according to the endpoint and consumption scenario. The calculated MOEs represent Pb exposure attributable to HCT alone and do not include exposure from other foods, drinking water, air, soil, or dust. They should therefore be interpreted as estimates of the incremental contribution of HCT consumption rather than as a complete characterization of total Pb exposure. Nevertheless, the occurrence of MOE values below 1, particularly under the medium- and high-consumption scenarios, supports strengthened monitoring of retail HCT with relatively high Pb concentrations and further investigation of its supply sources.

## 4. Conclusions

This province-wide point-of-purchase survey revealed distinct element-specific contamination patterns in market-sold HCT rhizomes across Guizhou. Pb was the most widespread regulatory concern, exceeding the applicable Chinese maximum limit in 87.1% of the 101 market-level composite samples, followed by Cd with an exceedance frequency of 49.5%. Hg exceeded its limit in only 4.0% of samples, while no exceedances were observed for As or Cr. The weak FDR-adjusted correlations and exploratory PCA indicated limited multi-element co-occurrence rather than a single uniform contamination pattern. Because production origins were not fully traceable, the observed geographic variation should be interpreted as point-of-purchase patterns rather than direct evidence of contamination in local production areas.

The five-element comparative screening HI increased with HCT consumption. The number of samples with HI > 1 increased from one under low consumption to three under medium consumption and 17 under high consumption. Cd and Pb were the principal contributors, accounting for mean sample-level contributions of 55.5% and 29.0%, respectively, and together representing 84.5% of the HI. Although As made a relatively small contribution in most samples, its 95th-percentile contribution reached 49.5%, showing that isolated high-As samples influenced the upper tail. Under high consumption, elevated HIs were more broadly distributed in Qianxinan and Bijie, whereas the higher prefecture-level means in Anshun and Qiandongnan were strongly affected by individual high-value samples.

Because the Pb THQ was calculated using a historical normalization benchmark, the five-element HI was interpreted only as a comparative screening indicator. The supplementary MOE assessment showed that the proportion of samples with renal MOE < 1 increased from 1.0% under low consumption to 16.8% and 32.7% under medium and high consumption, respectively; the corresponding proportions for the cardiovascular endpoint were 0%, 4.0%, and 12.9%. These results confirm that increasing HCT consumption progressively narrows the margin between Pb exposure and the selected effect-based reference points.

The findings identify market-sold HCT as a relevant dietary pathway for Cd and Pb exposure in Guizhou. Risk management should prioritize Pb regulatory compliance, the substantial contribution of Cd to the integrated HI, markets with repeated HI exceedances, and isolated samples with elevated As. Repeated retail monitoring should be combined with production-origin tracing and supply-chain investigation, while future assessments should incorporate element speciation, bioaccessibility, and the effects of washing and cooking.

## Figures and Tables

**Figure 1:**
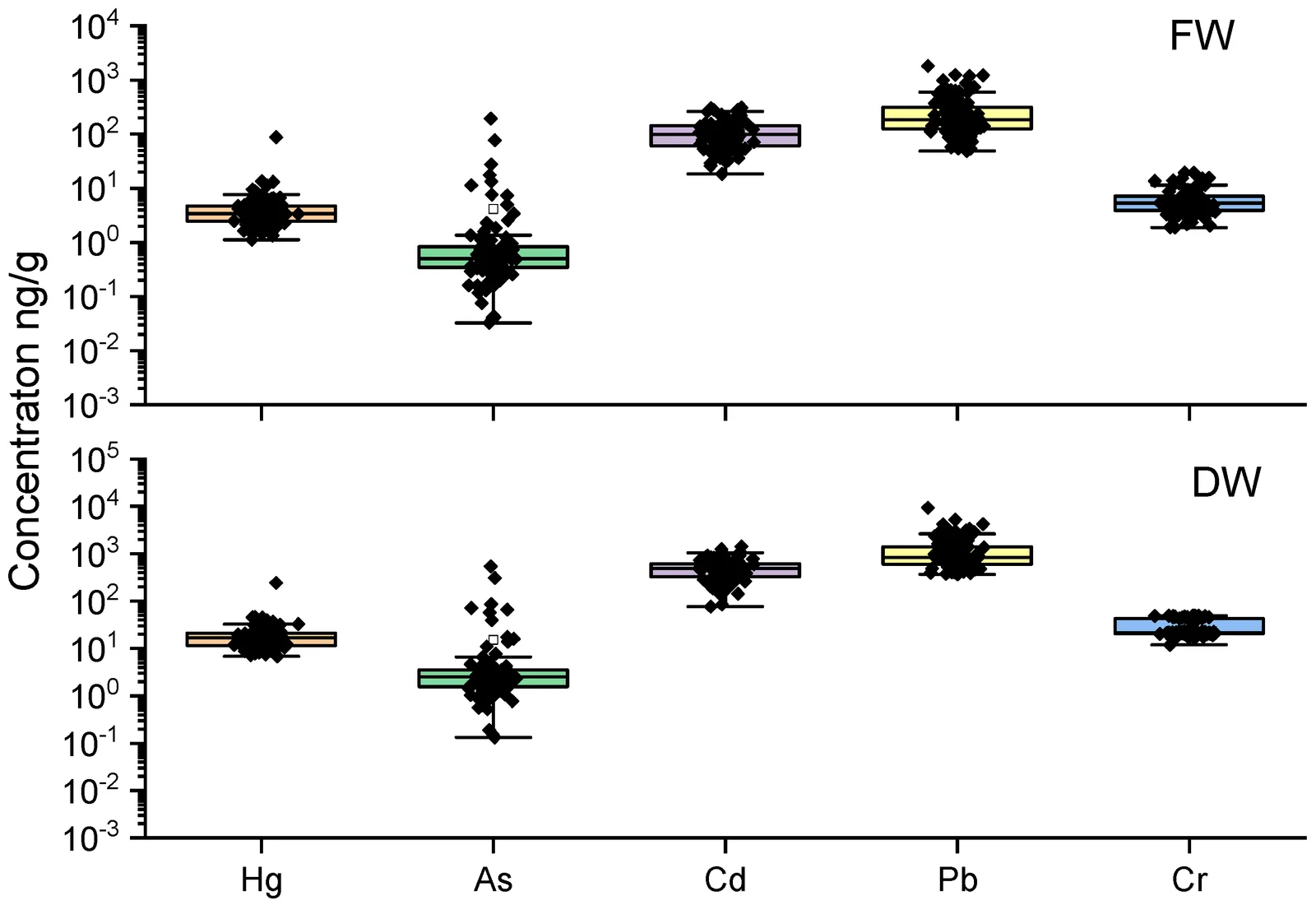
Mean Concentrations of As, Cd, Pb, Cr, and Hg in HCT from Guizhou, expressed on a dry weight (DW) and fresh weight (FW) basis.

**Figure 2:**
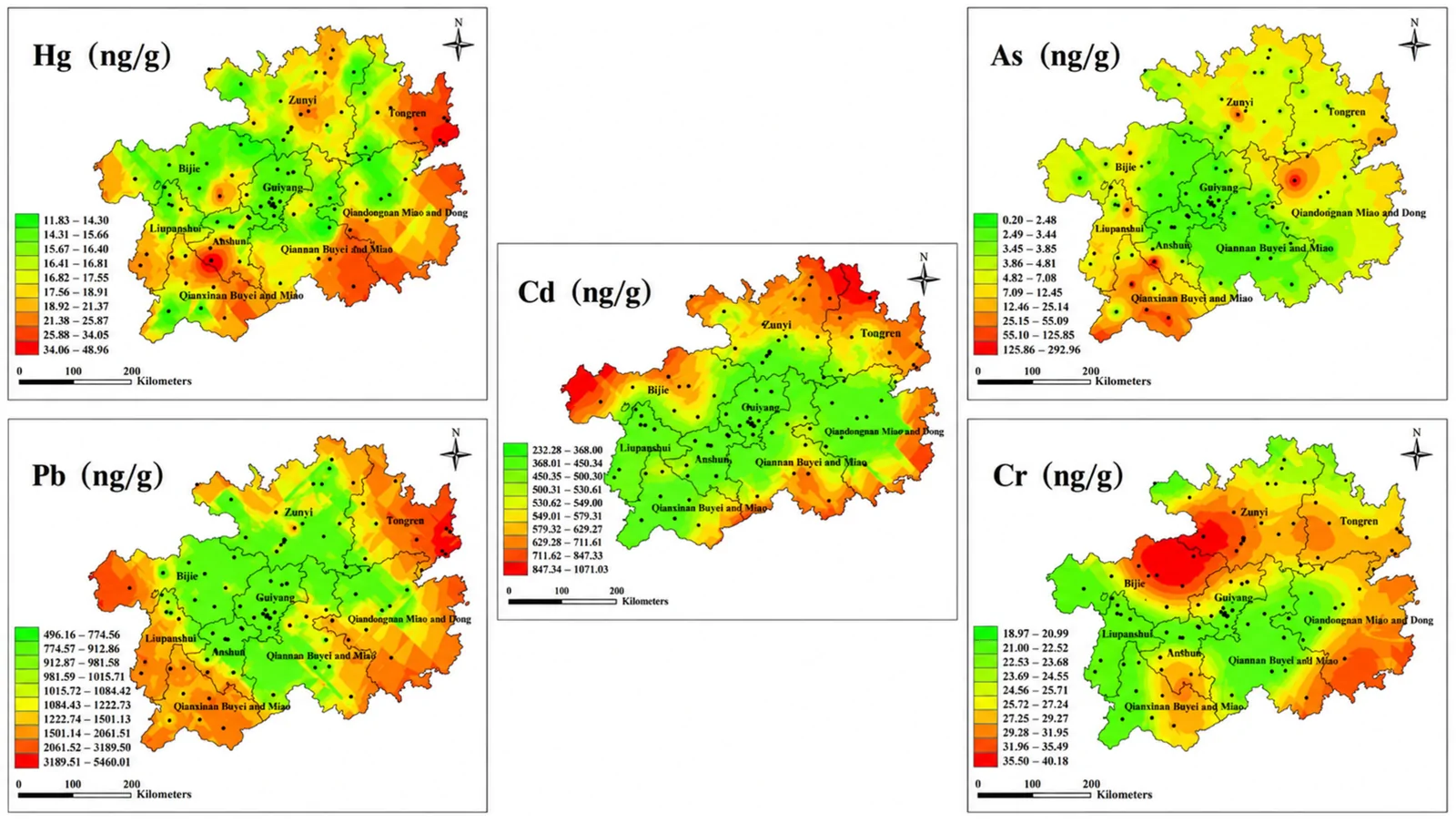
Kriging-interpolated point-of-purchase patterns of Hg, As, Cd, Pb, and Cr concentrations in HCT rhizomes obtained from retail markets across Guizhou, China. The surfaces were generated from 101 market-level composite samples and represent retail point-of-purchase variation rather than cultivation-site contamination. Sampling locations and coordinates are provided in Supplementary Table S3.

**Figure 3:**
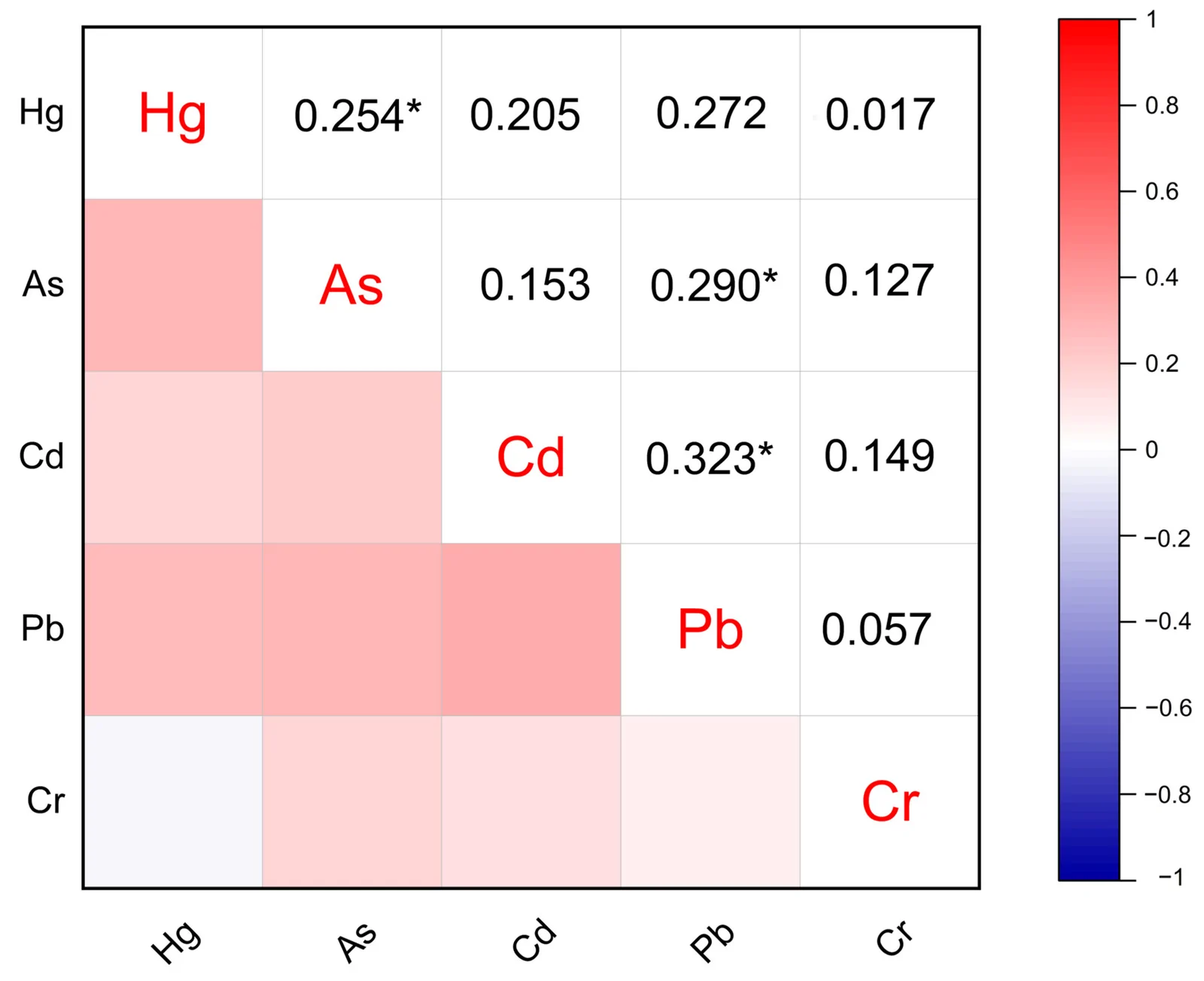
Spearman rank correlation matrix of Hg, As, Cd, Pb, and Cr concentrations in 101 market-level composite samples of HCT rhizomes obtained from retail markets across Guizhou, China. Values represent Spearman’s rank correlation coefficients (ρ). Statistical significance was determined using Benjamini–Hochberg false discovery rate-adjusted probabilities: * q < 0.05.

**Figure 4:**
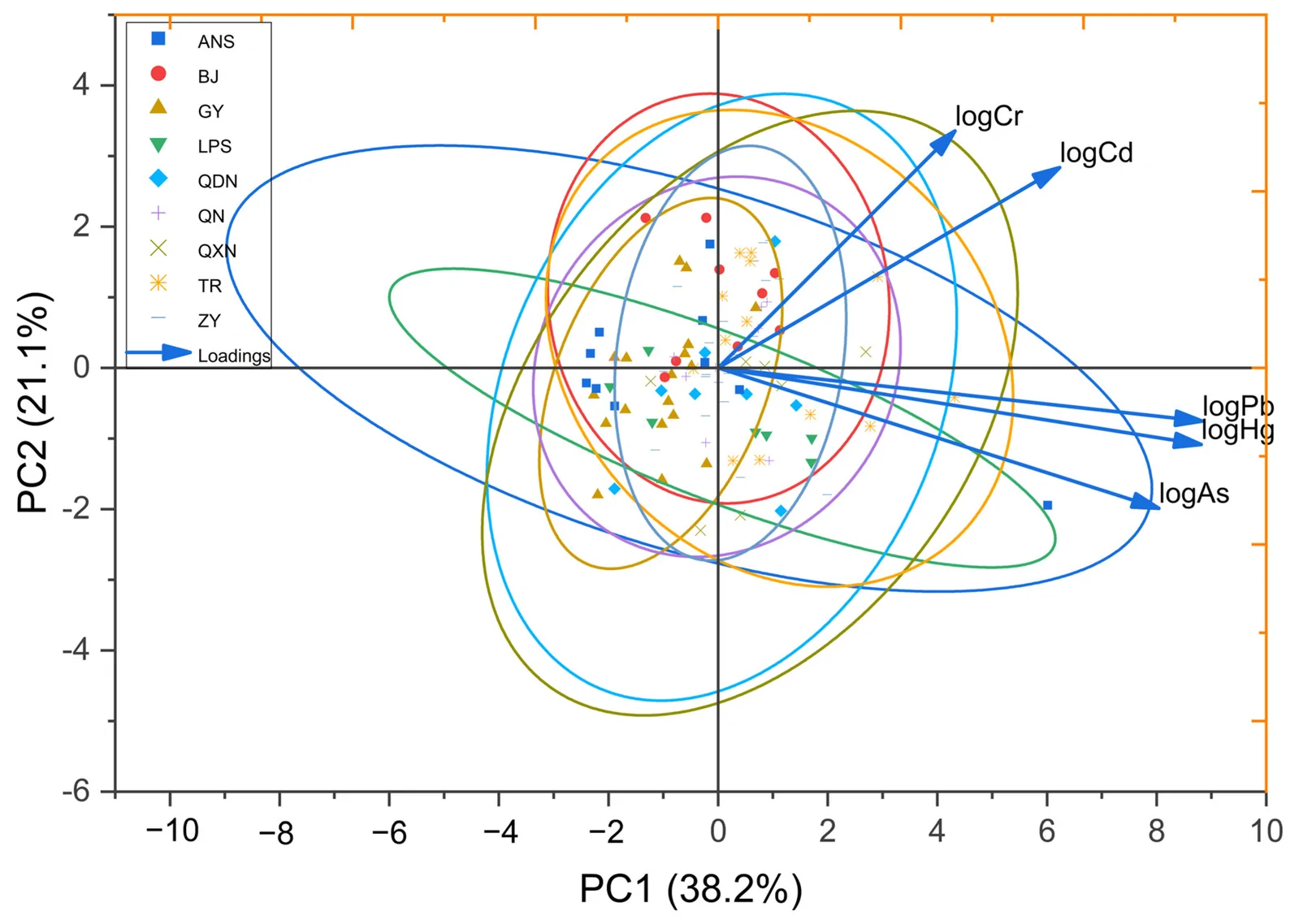
Principal component biplot based on log_10_-transformed and Z-score-standardized dry-weight concentrations of Hg, As, Cd, Pb, and Cr in market-level composite samples of HCT rhizomes. Points represent individual retail-market samples and are grouped by prefecture-level region. Arrows represent variable loadings on PC1 and PC2. The ellipses are descriptive 95% data ellipses and do not represent statistically distinct contamination groups. GZ: Guizhou; GY: Guiyang; ZY: Zunyi; TR: Tongren; QDN: Qiandongnan; LPS: Liupanshui; BJ: Bijie; ANS: Anshun; QN: Qiannan; QXN: Qianxinan.

**Figure 5:**
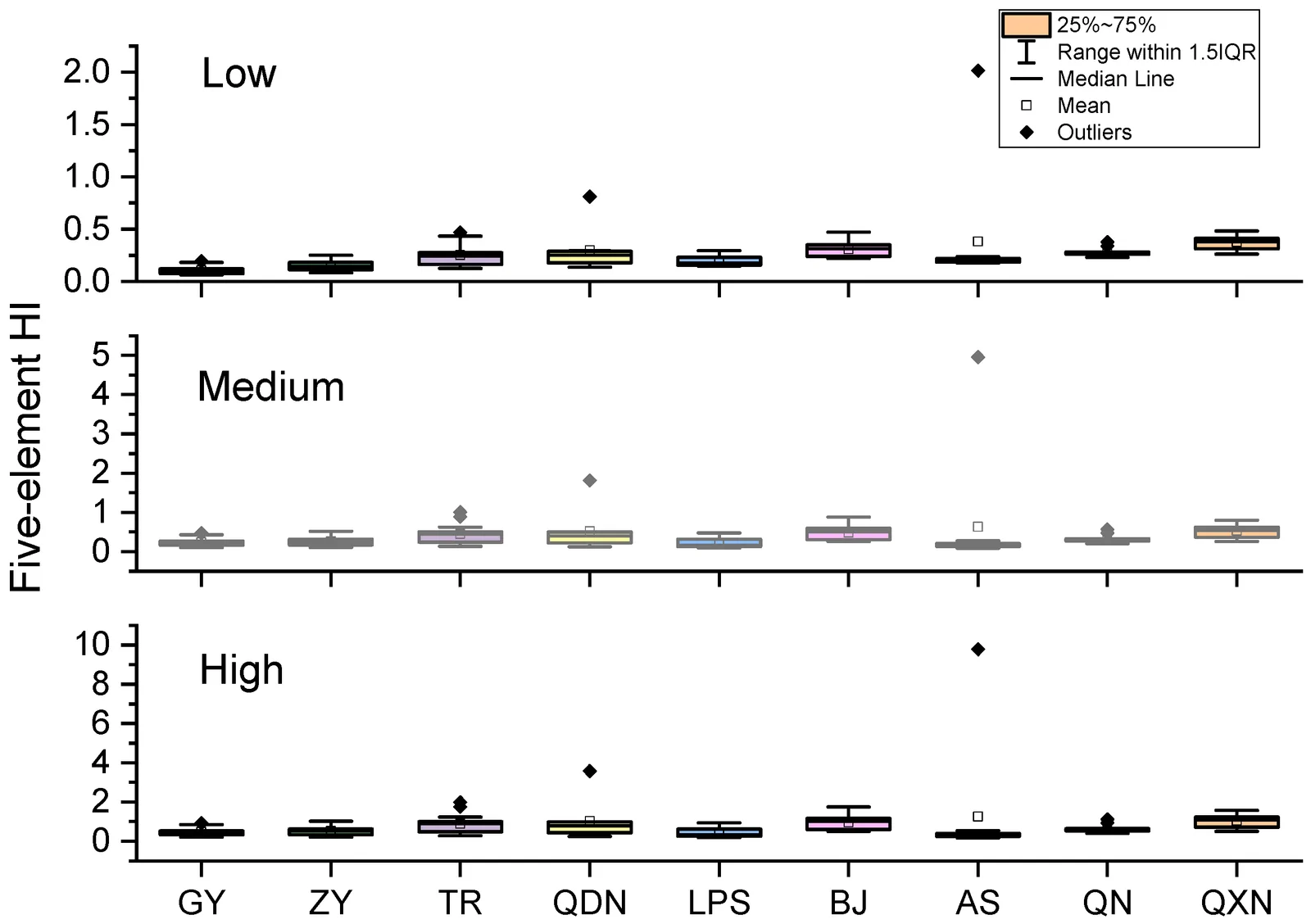
Prefecture-level distributions of the five-element hazard index under the low-, medium-, and high-HCT-consumption scenarios. Boxes represent the interquartile range, horizontal lines indicate medians, open squares indicate means, whiskers show values within 1.5 times the interquartile range, and solid diamonds indicate outliers. GY, Guiyang; ZY, Zunyi; TR, Tongren; QDN, Qiandongnan; LPS, Liupanshui; BJ, Bijie; AS, Anshun; QN, Qiannan; and QXN, Qianxinan.

**Table 1:** Overall distribution of the five-element hazard index under the three HCT-consumption scenarios.

Consumption Scenario	Mean ± SD	Median	IQR (Q1–Q3)	P95	Maximum	HI > 1, n (%)
Low	0.235 ± 0.213	0.200	0.137–0.270	0.433	2.017	1 (1.0%)
Medium	0.381 ± 0.517	0.274	0.179–0.448	0.793	4.958	3 (3.0%)
High	0.751 ± 1.021	0.542	0.353–0.883	1.566	9.786	17 (16.8%)

**Table 2:** Element-specific THQs and contributions to the five-element comparative screening HI.

Element	Median THQ, Low	Median THQ, Medium	Median THQ, High	Mean Contribution to HI (%)	Median Contribution, % (IQR)	THQ > 1, Low/Medium/High
As	0.0042	0.0101	0.0191	9.1	4.0 (2.7–6.7)	1/2/3
Cd	0.0603	0.1527	0.3013	55.5	59.4 (47.6–69.8)	0/0/0
Pb *	0.0250	0.0634	0.1251	29.0	25.3 (19.5–35.0)	0/0/1
Cr	0.0030	0.0076	0.0150	3.2	2.8 (1.8–4.2)	0/0/0
Hg	0.0030	0.0076	0.0150	3.2	2.8 (1.9–4.2)	0/0/0

Note: Element-specific contributions were calculated for each sample as THQ/HI ×100% and then summarized across the 101 samples. Because all element-specific quotients changed proportionally with ingestion rate, their percentage contributions were identical under the three consumption scenarios. * The Pb quotient was calculated using the historical normalization benchmark of 3.6 μg/(kg bw·day) and is included only as a component of the comparative screening HI.

**Table 3:** Pb margins of exposure for renal and cardiovascular endpoints under the three HCT-consumption scenarios.

Endpoint and Scenario	Mean ± SD	Median	IQR (Q1–Q3)	Minimum	MOE < 1, n (%)	1 ≤ MOE < 10, n (%)	MOE ≥ 10, n (%)
Renal, low	7.673 ± 5.161	6.993	3.997–9.286	0.692	1 (1.0%)	77 (76.2%)	23 (22.8%)
Renal, medium	3.029 ± 2.037	2.760	1.578–3.665	0.273	17 (16.8%)	83 (82.2%)	1 (1.0%)
Renal, high	1.535 ± 1.032	1.399	0.799–1.857	0.138	33 (32.7%)	68 (67.3%)	0 (0%)
Cardiovascular, low	18.270 ± 12.287	16.650	9.516–22.109	1.648	0 (0%)	26 (25.7%)	75 (74.3%)
Cardiovascular, medium	7.212 ± 4.850	6.572	3.756–8.727	0.650	4 (4.0%)	77 (76.2%)	20 (19.8%)
Cardiovascular, high	3.654 ± 2.457	3.330	1.903–4.422	0.330	13 (12.9%)	84 (83.2%)	4 (4.0%)

## Data Availability

The original contributions presented in this study are included in the article/Supplementary Material. Further inquiries can be directed to the corresponding author.

## Supplementary Materials

The following supporting information can be downloaded at: https://www.mdpi.com/article/10.3390/foods15193461/s1, Figure S1: Moisture content of *Houttuynia cordata* Thunb.(HCT) rhizomes across different regions of Guizhou Province; Table S1: Normality tests for the concentrations of five toxic elements; Table S2: Spearman rank correlations and Benjamini–Hochberg FDR-adjusted probabilities among five toxic elements in HCT rhizomes; Table S3: Sampling information for retail HCT rhizome samples collected across Guizhou Province; Table S4: Semivariogram parameters and cross-validation statistics for Kriging interpolation of toxic-element concentrations in retail HCT samples.
