# Supplementary material for: Point-of-Purchase Patterns of Cadmium, Lead, and Other Toxic Elements in Houttuynia cordata Thunb. Rhizomes from Retail Markets Across Guizhou, China
Source: Foods. 2026 Sep 28;15(19):3461. doi: 10.3390/foods15193461 (PMC13664355; doi:10.3390/foods15193461)
Supplement: Supplementary file 1 [file foods-15-03461-s001.zip › foods-4509391-supplementary.pdf]

## Supplementary Information

# Point-of-Purchase Patterns of Cadmium, Lead, and Other Toxic Elements in *Houttuynia cordata* Thunb. Rhizomes from Retail Markets Across Guizhou, China

Qingfeng Wang [1,*], Xuefeng He [2], Jia Xu [2], Dan Wang [1], Enguo Sheng [1], Bin Liu [1] and Zhonggen Li [1]

[1] School of Ecology, Zunyi Normal College, Pinan Road, Xinpu Town, Zunyi 563002, China;

lizhonggencn@126.com (Z.L.)

[2] Zunyi Ecological Environment Monitoring Center, No. 9, Sanxian North Road, Xinpu Town,

Zunyi 563002, China

* Correspondence: qfwang@zync.edu.cn

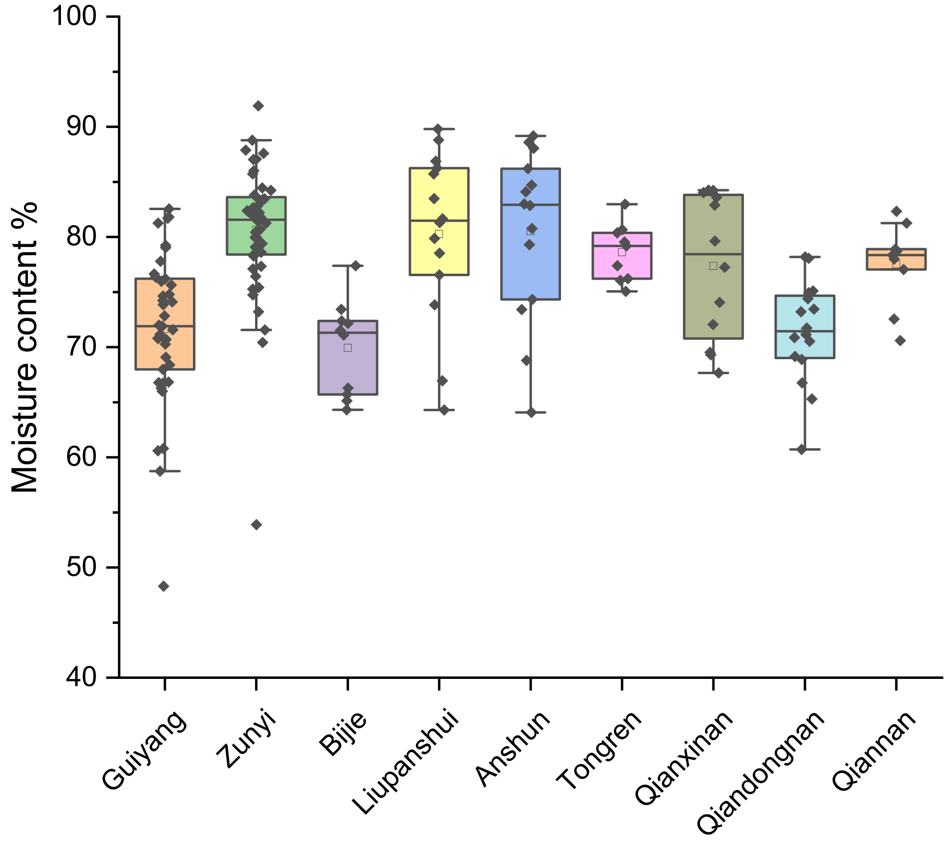


Figure S1 Moisture content of *Houttuynia cordata* Thunb.(HCT) rhizomes across different regions of Guizhou Province.

Table S1. Normality tests for the concentrations of five toxic elements

| **Element** | **Raw-data W** | **Raw-data p** | **$Log_{10}$-transformed W** | **$Log_{10}$-transformed p** |
|---|---|---|---|---|
| Hg | 0.3217 | $1.73 \times 10^{-19}$ | 0.9048 | $2.16 \times 10^{-6}$ |
| As | 0.2196 | $9.81 \times 10^{-21}$ | 0.8314 | $2.27 \times 10^{-9}$ |
| Cd | 0.9504 | $8.25 \times 10^{-4}$ | 0.9743 | 0.0458 |
| Pb | 0.6492 | $3.45 \times 10^{-14}$ | 0.9400 | $1.78 \times 10^{-4}$ |
| Cr | 0.6958 | $3.65 \times 10^{-13}$ | 0.7508 | $8.43 \times 10^{-12}$ |

Table S2. Spearman rank correlations and Benjamini–Hochberg FDR-adjusted probabilities among five toxic elements in HCT rhizomes

| **Element pair** | **Spearman's ρ** | **Raw p value** | **FDR-adjusted q value** | **Significance after FDR correction** | **Strength and direction** |
|---|---|---|---|---|---|
| Hg–As | 0.254 | 0.01046 | 0.02614 | Significant (*) | Weak positive |
| Hg–Cd | 0.205 | 0.03973 | 0.07945 | Not significant | Weak positive |

| | | | | | |
|---|---|---|---|---|---|
| Hg–Pb | 0.272 | 0.00585 | 0.01949 | Significant (*) | Weak positive |
| Hg–Cr | 0.017 | 0.86757 | 0.86757 | Not significant | Negligible |
| As–Cd | 0.153 | 0.12635 | 0.19574 | Not significant | Weak positive |
| As–Pb | 0.290 | 0.00321 | 0.01607 | Significant (*) | Weak positive |
| As–Cr | 0.127 | 0.20388 | 0.25485 | Not significant | Weak positive |
| Cd–Pb | 0.323 | 0.00098 | 0.00984 | Significant (**) | Weak positive |
| Cd–Cr | 0.149 | 0.13702 | 0.19574 | Not significant | Weak positive |
| Pb–Cr | 0.057 | 0.57127 | 0.63474 | Not significant | Negligible |

Table S3. Sampling information for retail HCT rhizome samples collected across Guizhou Province

| Sample ID | Prefecture-level area | County/District | Market type | Sampling period | Vendors pooled (n) | Longitude (°E) | Latitude (°N) |
|---|---|---|---|---|---|---|---|
| 1-1 | Guiyang | Huaxi District | Retail market | 18–23 July 2023 | 3 | 106.677465 | 26.424897 |
| 1-2 | Guiyang | Xiuwen County | Retail market | 18–23 July 2023 | 3 | 106.604810 | 26.844034 |
| 1-3 | Guiyang | Xiuwen County | Retail market | 18–23 July 2023 | 3 | 106.599926 | 26.836022 |
| 1-4 | Guiyang | Yunyan District | Retail market | 18–23 July 2023 | 3 | 106.663617 | 26.600072 |
| 1-5 | Guiyang | Yunyan District | Retail market | 18–23 July 2023 | 3 | 106.644618 | 26.573212 |
| 1-6 | Guiyang | Nanming District | Retail market | 18–23 July 2023 | 3 | 106.708632 | 26.557058 |
| 1-7 | Guiyang | Nanming District | Retail market | 18–23 July 2023 | 3 | 106.690046 | 26.550268 |
| 1-8 | Guiyang | Qingzhen City | Retail market | 18–23 July 2023 | 3 | 106.464565 | 26.556272 |
| 1-9 | Guiyang | Qingzhen City | Retail market | 18–23 July 2023 | 3 | 106.267151 | 26.728305 |
| 1-10 | Guiyang | Wudang District | Retail market | 18–23 July 2023 | 3 | 106.776581 | 26.639167 |
| 1-11 | Guiyang | Wudang District | Retail market | 18–23 July 2023 | 3 | 106.767942 | 26.630453 |

| Sample ID | Prefecture-level area | County/ District | Market type | Sampling period | Vendors pooled (n) | Longitude (°E) | Latitude (°N) |
|---|---|---|---|---|---|---|---|
| 1-12 | Guiyang | Guanshanhu District | Retail market | 18–23 July 2023 | 3 | 106.598714 | 26.613691 |
| 1-13 | Guiyang | Guanshanhu District | Retail market | 18–23 July 2023 | 3 | 106.598034 | 26.616151 |
| 1-14 | Guiyang | Baiyun District | Retail market | 18–23 July 2023 | 3 | 106.635153 | 26.676922 |
| 1-15 | Guiyang | Kaiyang County | Retail market | 18–23 July 2023 | 3 | 106.891795 | 27.039607 |
| 1-16 | Guiyang | Kaiyang County | Retail market | 18–23 July 2023 | 3 | 106.972977 | 27.058044 |
| 1-17 | Guiyang | Kaiyang County | Retail market | 18–23 July 2023 | 3 | 106.967468 | 27.061701 |
| 1-18 | Guiyang | Xifeng County | Retail market | 18–23 July 2023 | 3 | 106.733950 | 27.092373 |
| 2-1 | Zunyi | Huichuan District | Retail market | 16–19 April 2023 | 3 | 106.946459 | 27.712323 |
| 2-2 | Zunyi | Huichuan District | Retail market | 16–19 April 2023 | 3 | 106.934450 | 27.722553 |
| 2-3 | Zunyi | Honghuagang District | Retail market | 16–19 April 2023 | 3 | 106.933065 | 27.683656 |
| 2-4 | Zunyi | Honghuagang District | Retail market | 16–19 April 2023 | 3 | 106.893199 | 27.644725 |
| 2-5 | Zunyi | Bozhou District | Retail market | 16–19 April 2023 | 3 | 106.828701 | 27.535281 |
| 2-6 | Zunyi | Meitan County | Retail market | 16–19 April 2023 | 3 | 107.482952 | 27.765325 |
| 2-7 | Zunyi | Fenggang County | Retail market | 16–19 April 2023 | 3 | 107.717704 | 27.953388 |
| 2-8 | Zunyi | Daozhen County | Retail market | 16–19 April 2023 | 3 | 107.607727 | 28.885240 |
| 2-9 | Zunyi | Daozhen County | Retail market | 16–19 April 2023 | 3 | 107.571351 | 28.763662 |
| 2-10 | Zunyi | Xishui County | Retail market | 16–19 April 2023 | 3 | 106.204440 | 28.368078 |
| 2-11 | Zunyi | Suiyang County | Retail market | 16–19 April 2023 | 3 | 107.084647 | 27.897783 |
| 2-12 | Zunyi | Suiyang | Retail | 16–19 April 2023 | 3 | 107.193081 | 27.955898 |

| Sample ID | Prefecture-level area | County/ District | Market type | Sampling period | Vendors pooled (n) | Longitude (°E) | Latitude (°N) |
|---|---|---|---|---|---|---|---|
| | | County | market | | | | |
| 2-13 | Zunyi | Yuqing County | Retail market | 16–19 April 2023 | 3 | 107.811683 | 27.313451 |
| 2-14 | Zunyi | Yuqing County | Retail market | 16–19 April 2023 | 3 | 108.134564 | 27.266075 |
| 2-15 | Zunyi | Renhuai City | Retail market | 16–19 April 2023 | 3 | 106.390011 | 27.765428 |
| 2-16 | Zunyi | Wuchuan County | Retail market | 16–19 April 2023 | 3 | 107.899493 | 28.549626 |
| 2-17 | Zunyi | Zhengan County | Retail market | 16–19 April 2023 | 3 | 107.460402 | 28.540273 |
| 2-18 | Zunyi | Zhengan County | Retail market | 16–19 April 2023 | 3 | 107.343626 | 28.545097 |
| 2-19 | Zunyi | Tongzi County | Retail market | 16–19 April 2023 | 3 | 106.828056 | 28.136309 |
| 2-20 | Zunyi | Chishui City | Retail market | 16–19 April 2023 | 3 | 105.696102 | 28.590754 |
| 3-1 | Tongren | Dejiang County | Retail market | 5–7 August 2023 | 3 | 108.059777 | 28.246692 |
| 3-2 | Tongren | Yanhe County | Retail market | 5–7 August 2023 | 3 | 108.503435 | 28.564127 |
| 3-3 | Tongren | Sinan County | Retail market | 5–7 August 2023 | 3 | 108.257003 | 27.944301 |
| 3-4 | Tongren | Songtao County | Retail market | 5–7 August 2023 | 3 | 109.207321 | 28.160673 |
| 3-5 | Tongren | Shiqian County | Retail market | 5–7 August 2023 | 3 | 108.218170 | 27.502529 |
| 3-6 | Tongren | Shiqian County | Retail market | 5–7 August 2023 | 3 | 108.081042 | 27.428343 |
| 3-7 | Tongren | Yuping County | Retail market | 5–7 August 2023 | 3 | 108.912941 | 27.246201 |
| 3-8 | Tongren | Wanshan District | Retail market | 5–7 August 2023 | 3 | 109.197331 | 27.519013 |
| 3-9 | Tongren | Wanshan District | Retail market | 5–7 August 2023 | 3 | 109.240894 | 27.477539 |
| 3-10 | Tongren | Jiangkou County | Retail market | 5–7 August 2023 | 3 | 108.836120 | 27.711093 |
| 3-11 | Tongren | Bijiang District | Retail market | 5–7 August 2023 | 3 | 109.287660 | 27.813286 |

| Sample ID | Prefecture-level area | County/ District | Market type | Sampling period | Vendors pooled (n) | Longitude (°E) | Latitude (°N) |
|---|---|---|---|---|---|---|---|
| 3-12 | Tongren | Bijiang District | Retail market | 5–7 August 2023 | 3 | 109.245039 | 27.851510 |
| 3-13 | Tongren | Yinjiang County | Retail market | 5–7 August 2023 | 3 | 108.440802 | 28.022689 |
| 4-1 | Qiandongnan | Leishan County | Retail market | 19–21 August 2023 | 3 | 108.077574 | 26.378322 |
| 4-2 | Qiandongnan | Taijiang County | Retail market | 19–21 August 2023 | 3 | 108.319775 | 26.668584 |
| 4-3 | Qiandongnan | Jianhe County | Retail market | 19–21 August 2023 | 3 | 108.436608 | 26.727570 |
| 4-4 | Qiandongnan | Sansui County | Retail market | 19–21 August 2023 | 3 | 108.697361 | 26.957752 |
| 4-5 | Qiandongnan | Danzhai County | Retail market | 19–21 August 2023 | 3 | 107.815729 | 26.233345 |
| 4-6 | Qiandongnan | Huangping County | Retail market | 19–21 August 2023 | 3 | 107.937856 | 26.907424 |
| 4-7 | Qiandongnan | Majiang County | Retail market | 19–21 August 2023 | 3 | 107.595914 | 26.518527 |
| 4-8 | Qiandongnan | Rongjiang County | Retail market | 19–21 August 2023 | 3 | 108.469450 | 25.954967 |
| 5-1 | Liupanshui | Zhongshan District | Retail market | 29 July and 5 August 2023 | 3 | 104.803902 | 26.776373 |
| 5-2 | Liupanshui | Zhongshan District | Retail market | 29 July and 5 August 2023 | 3 | 104.887613 | 26.670755 |
| 5-3 | Liupanshui | Liuzhi Special District | Retail market | 29 July and 5 August 2023 | 3 | 105.090122 | 26.472177 |
| 5-4 | Liupanshui | Panzhou City | Retail market | 29 July and 5 August 2023 | 3 | 104.468638 | 25.707882 |
| 5-5 | Liupanshui | Panzhou City | Retail market | 29 July and 5 August 2023 | 3 | 104.556333 | 25.853464 |
| 5-6 | Liupanshui | Shuicheng District | Retail market | 29 July and 5 August 2023 | 3 | 104.875246 | 26.535710 |
| 5-7 | Liupanshui | Shuicheng District | Retail market | 29 July and 5 August 2023 | 3 | 104.946763 | 26.530823 |
| 6-1 | Bijie | Qianxi City | Retail market | 22–23 August 2023 | 3 | 106.029971 | 27.006816 |
| 6-2 | Bijie | Dafang County | Retail market | 22–23 August 2023 | 3 | 105.628576 | 27.153324 |

| Sample ID | Prefecture-level area | County/ District | Market type | Sampling period | Vendors pooled (n) | Longitude (°E) | Latitude (°N) |
|---|---|---|---|---|---|---|---|
| 6-3 | Bijie | Nayong County | Retail market | 22–23 August 2023 | 3 | 105.409070 | 26.776610 |
| 6-4 | Bijie | Hezhang County | Retail market | 22–23 August 2023 | 3 | 104.754476 | 27.124146 |
| 6-5 | Bijie | Jinhaihu District | Retail market | 22–23 August 2023 | 3 | 105.481792 | 27.141333 |
| 6-6 | Bijie | Zhijin County | Retail market | 22–23 August 2023 | 3 | 105.771617 | 26.678298 |
| 6-7 | Bijie | Weining County | Retail market | 22–23 August 2023 | 3 | 104.262473 | 26.889174 |
| 6-8 | Bijie | Qixingguan District | Retail market | 22–23 August 2023 | 3 | 105.303100 | 27.297658 |
| 6-9 | Bijie | Jinsha County | Retail market | 22–23 August 2023 | 3 | 106.219480 | 27.470821 |
| 7-1 | Anshun | Puding County | Retail market | 15–17 August 2023 | 3 | 105.738628 | 26.299837 |
| 7-2 | Anshun | Ziyun County | Retail market | 15–17 August 2023 | 3 | 106.079451 | 25.752480 |
| 7-3 | Anshun | Guanling County | Retail market | 15–17 August 2023 | 3 | 105.616129 | 25.752121 |
| 7-4 | Anshun | Guanling County | Retail market | 15–17 August 2023 | 3 | 105.601014 | 25.923804 |
| 7-5 | Anshun | Pingba District | Retail market | 15–17 August 2023 | 3 | 106.267484 | 26.409322 |
| 7-6 | Anshun | Pingba District | Retail market | 15–17 August 2023 | 3 | 106.284663 | 26.393973 |
| 7-7 | Anshun | Xixiu District | Retail market | 15–17 August 2023 | 3 | 105.940383 | 26.240616 |
| 7-8 | Anshun | Xixiu District | Retail market | 15–17 August 2023 | 3 | 105.989962 | 26.234578 |
| 7-9 | Anshun | Zhenning County | Retail market | 15–17 August 2023 | 3 | 105.764717 | 26.055977 |
| 7-10 | Anshun | Zhenning County | Retail market | 15–17 August 2023 | 3 | 105.834671 | 26.049714 |
| 8-1 | Qiannan | Duyun City | Retail market | 7–11 August 2023 | 3 | 107.517771 | 26.260440 |
| 8-2 | Qiannan | Fuquan City | Retail market | 7–11 August 2023 | 3 | 107.517796 | 26.688103 |
| 8-3 | Qiannan | Pingtang | Retail | 7–11 August 2023 | 3 | 107.339328 | 25.829039 |

| Sample ID | Prefecture-level area | County/ District | Market type | Sampling period | Vendors pooled (n) | Longitude (°E) | Latitude (°N) |
|---|---|---|---|---|---|---|---|
| | | County | market | | | | |
| 8-4 | Qiannan | Guiding County | Retail market | 7–11 August 2023 | 3 | 107.232101 | 26.585046 |
| 8-5 | Qiannan | Luodian County | Retail market | 7–11 August 2023 | 3 | 106.751750 | 25.425531 |
| 8-6 | Qiannan | Dushan County | Retail market | 7–11 August 2023 | 3 | 107.551982 | 25.844863 |
| 8-7 | Qiannan | Sandu County | Retail market | 7–11 August 2023 | 3 | 107.885612 | 25.983066 |
| 8-8 | Qiannan | Longli County | Retail market | 7–11 August 2023 | 3 | 106.979668 | 26.453441 |
| 8-9 | Qiannan | Libo County | Retail market | 7–11 August 2023 | 3 | 107.886729 | 25.410758 |
| 9-1 | Qianxinan | Xingyi City | Retail market | 27–28 August 2023 | 3 | 104.921236 | 25.055754 |
| 9-2 | Qianxinan | Ceheng County | Retail market | 27–28 August 2023 | 3 | 105.810631 | 24.984318 |
| 9-3 | Qianxinan | Puan County | Retail market | 27–28 August 2023 | 3 | 104.952932 | 25.783061 |
| 9-4 | Qianxinan | Zhenfeng County | Retail market | 27–28 August 2023 | 3 | 105.649814 | 25.386877 |
| 9-5 | Qianxinan | Qinglong County | Retail market | 27–28 August 2023 | 3 | 105.218458 | 25.835107 |
| 9-6 | Qianxinan | Xingren City | Retail market | 27–28 August 2023 | 3 | 105.185502 | 25.435447 |
| 9-7 | Qianxinan | Anlong County | Retail market | 27–28 August 2023 | 3 | 105.438504 | 25.097763 |

**Table S4. Semivariogram parameters and cross-validation statistics for Kriging interpolation of toxic-element concentrations in retail HCT samples**

| Element | Transformation[a] | Kriging method | Model | Nugget | Range | Sill | Nugget/Sill (%) | Kriging ME | Kriging MAE | Kriging RMSE | Mean standardized error | RMS standardized error | IDW ME | IDW MAE | IDW RMSE |
|---|---|---|---|---|---|---|---|---|---|---|---|---|---|---|---|
| Cd | Natural logarithm | Simple Kriging | Exponential | 0.2140 | 0.1123 | 0.9765 | 21.9150 | 0.057 | 0.752 | 0.964 | 0.073 | 1.1321 | 0.084 | 0.808 | 1.048 |
| Pb | Natural logarithm | Ordinary Kriging | Stable | 53.2536 | 0.0547 | 234.5214 | 22.7073 | 0.007 | 0.817 | 1.068 | 0.0019 | 1.1425 | 0.025 | 0.897 | 1.163 |
| As | Natural logarithm | Simple Kriging | Stable | 0.0001 | 0.0425 | 1.0247 | 0.0098 | −0.013 | 0.661 | 0.847 | −0.0142 | 0.9418 | −0.027 | 0.718 | 0.928 |
| Cr | Natural logarithm | Simple Kriging | Exponential | 0.1231 | 0.0513 | 0.9997 | 12.3137 | −0.009 | 0.745 | 0.954 | −0.0081 | 1.1102 | −0.029 | 0.809 | 1.036 |
| Hg | Natural logarithm | Simple Kriging | Stable | 0.3054 | 0.0832 | 0.9999 | 30.5431 | 0.023 | 0.692 | 0.893 | 0.0253 | 0.9047 | 0.037 | 0.747 | 0.963 |
